# A novel treatment approach to infected nonunion of long bones without systemic antibiotics

**DOI:** 10.1007/s11751-018-0303-4

**Published:** 2018-01-29

**Authors:** Karim Z. Masrouha, Michael E. Raad, Said S. Saghieh

**Affiliations:** Department of Surgery, Orthopedics Division, American University of Beirut Medical Center, P. O. Box 11-0236, Riad El-Solh, Beirut, 1107 2020 Lebanon

**Keywords:** Infection, Nonunion, Local antibiotics, Calcium sulphate, Bone pellet, Long bones

## Abstract

Infected nonunion of long bones may require intravenous antibiotics over a lengthy period which may result in a high rate of complications. This study aims to assess the efficacy of local antibiotics used as a replacement to prolonged intravenous therapy. Thirteen patients with infected nonunion of long bones who failed at least one previous surgery were included. The infection was treated through extensive debridement, application of antibiotic-impregnated calcium sulphate pellets and the bone stabilized with external fixation. These patients were monitored for union and infection by clinical signs, laboratory values, and radiographs over a period of 24 months. The results support an eradication of infection and union in all patients with no antibiotic-associated complications. Local antibiotic delivery using calcium sulphate pellets provides an effective method for treatment of nonunion in long bones and is free of the complications from the intravenous route.

## Introduction

Infected nonunion is a dreaded complication of the long bone fractures because its management is challenging to both the patient and the orthopaedic surgeon [1]. Management involves surgical debridement, tissue reconstruction, a long-course of antibiotics and opioids. The complexity of care and its prolonged duration increases the cost of the treatment for the patient [2]. In addition, adverse sequelae may lead to bone loss, residual deformity, or even amputation [1].

Recent advances in surgical technique have tilted the balance towards limb salvage surgery [1]; however, only a few high-quality studies exist which describe the most beneficial and cost-effective post-operative antimicrobial treatment approach [3]. Currently, the most widely used approach is extensive surgical debridement followed by 6 weeks of high-dose intravenous (IV) antibiotics [4].

The effective antibiotic penetration and eradication of infection requires 10–100 times the minimum inhibitory concentration (MIC). This high dose is required due to the bacterial biofilm formation and the local milieu at the infection site [5]. When systemic antibiotics are administered for a prolonged period, there is a significant risk of complications. The IV route requires frequent replacement because it is associated with IV-line thrombosis, infection, and stenosis [6]. Other complications include, but are not limited to, haematologic, renal, dermatologic, gastrointestinal, and other metabolic problems such as lactic acidosis [7]. Certain antibiotics used in the treatment of osteomyelitis are considered more toxic than others, particularly aminoglycosides and vancomycin. These two antibiotics require frequent measurement of trough and creatinine levels, further contributing to the burden of treatment [7]. The high frequency of *Staphylococcus aureus* infection and its high rates of methicillin resistance [8] dictate the use of vancomycin as a first-line treatment. Although effective, vancomycin is associated with nephrotoxicity in as many as 43% of patients in some studies [9].

The use of a local antibiotic delivery conduit was popularized in Europe during the 1970s [5]. It is associated with lower rates of systemic antibiotic toxicity, allowed for filling of the defect created by extensive debridement, and was considerably cheaper than prolonged courses of systemic antibiotics [8]. Polymethyl-methacrylate (PMMA) is one of the most commonly used antibiotic conduits today but requires further surgery for removal and may be associated with bead infection after elution of the antibiotic has completed [10]. Recently, synthetic calcium sulphate has gained popularity as an attractive alternative due to its predictable drug-eluting properties, osteoconductivity, biodegradability and obviating additional surgery for removal [11].

The beneficial effects of this treatment modality necessitate further study to document its efficiency in the absence of systemic antibiotic use. The aim of our study is to evaluate the resolution of infection and bone healing in patients with infected nonunion of long bones treated with extensive debridement, bone reconstruction and antibiotic-impregnated calcium sulphate pellets without subsequent use of systemic antibiotics.

## Materials and methods

This is a retrospective cohort study of 13 consecutive patients with infected nonunion of long bone from two affiliated medical centres between 2003 and 2012. Institutional review board approval was obtained from both institutions prior to initiation of the study. All patients included in the study had stage IV osteomyelitis as per the Cierney–Mader classification system [12], failed at least one previous surgery, and were operated on by one surgeon. All patients were followed up post-operatively for a period of 24 months before being discharged from the clinic. Patients under the age of 18 and with infected nonunions other that long bones were excluded.

### Surgical procedure

Use of the bone graft substitute was an integral part of our protocol. Any previously implanted hardware was removed. Thorough debridement of all nonviable bone and necrotic soft tissue was performed. The site of infection was irrigated thoroughly with large quantities of Normal Saline. All surgically extracted tissues were sent for culture. The paprika sign was used to detect viable bone. An external fixator was then applied to hold the nonunion site. When needed, a remote osteotomy was performed for gradual distraction. Bone regeneration was planned dependent upon the bone loss and according to the principles of histogenesis [13]. Then, self-prepared calcium sulphate pellets were used to fill the defect (Stimulan Kit, Biocomposites Ltd., Staffordshire, England).

The paste was prepared according to the instructions in the supplied kit and mixed with 1 g of vancomycin and 240 mg of gentamicin. The result was a combination of pellets impregnated with antibiotics, measuring 3 mm and 4.8 mm in diameter.

### Outcomes measured

The study outcomes were union and the presence of infection. Bone union was defined as bridging of three out of four cortices on two orthogonal radiographs [14]. Infection was assessed by clinical signs (erythema, drainage, and wound problems) and further need for systemic antibiotics. These parameters were assessed during outpatient follow-up visits as part of the standard of care and biannually after discharge from the clinic.

Also, the erythrocyte sedimentation rate (ESR) was measured, as per the hospital’s protocol, and reported for each patient post-operatively. Normal ESR ranges between 0 and 15 mm/h for those younger than 50 and between 1 and 20 mm/h for those older than 50. Microbiological assays from specimens that were extracted intraoperatively were analysed. The mean and standard deviation (SD) were obtained for continuous data sets, and others were reported as modes.

## Results

### Patient characteristics

The average age was 35 years (18–63 years). All patients were male. The majority of structural defects involved the tibia; the femur and humerus were a lower proportion (Table 1). All patients had previously been operated on with an average of three surgeries (Table 1). Pre-operatively, nine patients had draining sinuses and four had evidence of skin necrosis with soft tissue defects (Table 1).

**Table 1:** Patient demographics and pre-operative characteristics

Patient	Age	Sex	Fracture site	Number of previous surgeries	Signs of infection
1	35	Male	Tibia	3	Soft tissue defect
2	18	Male	Femur	3	Draining sinus
3	50	Male	Tibia	9	Soft tissue defect
4	44	Male	Humerus	3	Draining sinus
5	28	Male	Tibia	5	Draining sinus
6	24	Male	Femur	2	Draining sinus
7	22	Male	Tibia	4	Draining sinus
8	25	Male	Tibia	3	Soft tissue defect
9	22	Male	Tibia	1	Draining sinus
10	43	Male	Humerus	1	Draining sinus
11	45	Male	Tibia	2	Draining sinus
12	63	Male	Tibia	3	Draining sinus
13	40	Male	Tibia	4	Soft tissue defect
Average	35.3 ± 13.5	Male	Tibia	3	Draining sinus

All averages indicate mode except age whose average is expressed as mean ± standard deviation

Eight patients underwent acute shortening with pellets implanted at the time of surgery. Five had bone transport with pellets implanted at the time of docking site grafting. All patients had the external fixator in place until bony union. One patient had to undergo bone grafting for nonunion at the docking site, which subsequently healed.

### Union

**Fig. 1:**
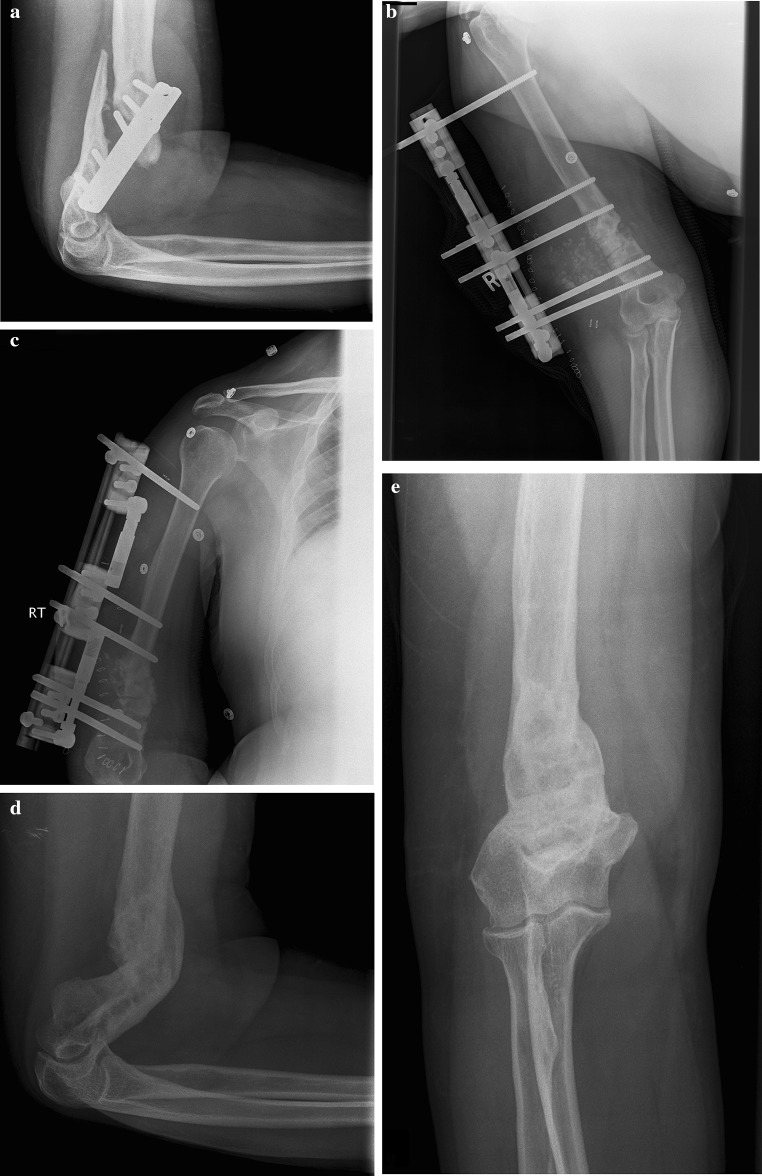
a Pre-operative radiograph of Patient 10 shows nonunion at the distal end of the humerus; **b** and **c** immediate post-operative radiographs showing the external fixator and calcium sulphate pellets in place; **d** and **e** follow-up anteroposterior and lateral radiograph shows the healed fracture

All patients achieved union during the follow-up period as evidenced on radiographs (Fig. 1). The average time to union was 5.5 months (3–11 months) (Table 2).

**Table 2:** Post-operative union and infection parameter outcomes

Patient	Time to union (months)	ESR post-operatively (mm/h)	Culture results	Resolution of clinical signs	Outpatient systemic antibiotic therapy
1	3	9	Mixed	Yes	None
2	11	11	Mixed	Yes	None
3	3	15	*Escherichia.coli (ESBL)*	Yes	None
4	4	5	*Staphylococcus* coagulase negative	Yes	None
5	4	7	*E. coli*	Yes	None
6	8	11	*Staphylococcus aureus*	Yes	None
7	11	4	*S. aureus*	Yes	None
8	6	8	*Staphylococcus* coagulase negative	Yes	None
9	3	13	Mixed	Yes	None
10	6	11	*Staphylococcus* coagulase negative	Yes	None
11	4	7	*Staphylococcus* coagulase Negative	Yes	None
12	6	12	*Eneterobacter cloacae*	Yes	None
13	3	15	*Staphylococcus* coagulase negative	Yes	None
Average	5.5 ± 2.9	9.8 ± 3.5	*Staphylococcus* coagulase negative	Yes	None

All averages indicate mode except time to union and erythrocyte sedimentation rate (ESR) whose averages are expressed as mean ± standard deviation. The mixed culture result included more than one of the following *Pseudomonas cepaciae*; *Klebsiella oxitocae*; *Pseudomonas aeroginosa*; *Staphylococcus aureus*; and/or coagulase-negative *Staphylococcus*

### Infection

The ESR was found to be normal in all 13 patients post-operatively with an average of 9.8 ± 3.5 mm/h (Table 2). All cases involving a draining sinus, or with local signs of infection such as erythema, warmth, swelling or pain over the treated segment resolved in the post-operative follow-up period. No recurrences were noted in that time (Table 2).

### Microbiology

Bone cultures were taken from all patients intraoperatively. Microbiological analyses showed coagulase-negative *Staphylococcus* (5 cases) to be the most common. The other culture results were as follows: *Staphylococcus aureus* (2 cases), *Escherichia Coli* (2 cases), *Enterobacter cloacae* (1 case), and three cases had mixed infections involving gram-negative bacilli and gram-positive cocci (Table 2).

### Need for systemic antimicrobial therapy after discharge

Patients received IV antibiotics immediately after debridement and during their hospital stay, which was typically three to 5 days. None of the patients received systemic antibiotics after being discharged from the hospital.

## Discussion

Our treatment modality eradicated infection and allowed for bone healing, while sparing patients prolonged courses of IV antibiotics. All of our patients achieved union after an average of 5.5 months and had no clinical signs of infection within the period of follow-up. None of the patients required IV antibiotics after leaving the hospital and the only antibiotic to which they were exposed was that impregnated in the calcium sulphate beads.

Local antibiotic beads allow the delivery of high antibiotic concentrations locally with very low levels in the systemic circulation [15]. Calcium sulphate offers the advantage of biodegradability over PMMA, sparing patients a second surgery for removal. Its osteoinductive properties also aided bone healing and filling the bone gap created by extensive debridement [16].

Our choice of surgical procedure was based on our own experience and a review by Peter et al. [17], in which 34 clinical trials for the treatment of infected nonunion of the long bones were studied. Although there is no consensus, the highest cure rates are observed with the two-stage procedure of debridement, antibiotic beads, and planned secondary fixations [17]. We believe that the surgical procedure is an integral part of the treatment protocol, and we emphasize the role of thorough debridement of any necrotic tissue before the application of beads. Selhi et al. [18] looked at a treatment protocol similar to ours but with PMMA beads applied to 16 patients. Thirteen per cent of the patients in their study failed to achieve union and eradication of infection. This was attributed to inadequate debridement and the choice of bone cement [18]. Calcium sulphate has been shown to be just as good or even superior to PMMA as a carrier of antibiotics to infected surgical sites [19], and our study supports the efficiency of calcium sulphate.

Another large series by Ferguson et al. looked at 195 patients treated with surgery and calcium sulphate pellets, followed by intravenous antibiotics for 6 weeks. After the initial treatment, 91.8% of patients achieved eradication of infection [20]. Higher rates of success were achieved in our study and without extended use of intravenous antibiotics. However, the average age in their study was 46.1 years (16.1–82 years), 11 years older than the average age in our study sample (35.3 years), and our oldest patient was 63 years old. Advancing age has a well-known impact on the regenerative capacity of bone [21] which might have influenced the success rate.

Gauland et al. [11] reported eradication of infection in 86.4% of 337 subjects treated with the same modality as ours. The lower rate might be partly explained by the difference in our inclusion criteria, which included patients with osteomyelitis of the lower extremity only. The foot, being a distant organ, is associated with poorer perfusion when compared to the long bones and much infective pathology in this area associated with diabetes which tips the balance in favour of continuing infection [22]. It is worth mentioning that the vancomycin dose mixed with the 10 mL calcium sulphate paste is higher in our series (1 g versus 500 mg). However, there is insufficient data in the literature regarding the effect of the antibiotic dose on treatment outcome for this treatment modality.

There is growing evidence for the idea that long-term intravenous antibiotics in infected nonunion does not guarantee a cure and is associated with high levels of antibiotic and catheter-related complications [23]. As such, the authors attribute the success of treatment to the aggressive debridement as well as the elution and bone healing properties of calcium sulphate bone substitute.

We acknowledge the limitations inherent in all retrospective cohorts. Although this study had a small sample size, it adds to the body of evidence of antibiotic-impregnated bone substitute treatment of patients with infected nonunion. We recommend further investigations through larger series and randomized control trials comparing it to different modalities of treatment. Although most recurrences of osteomyelitis occur within 2 years of treatment [24], it is well known to recur up to several decades later [22]. Therefore, a longer follow-up period will also allow for better evaluation of treatment outcomes.

## Conclusion

Based on our study, the use of antibiotic-impregnated calcium sulphate pellets without subsequent extended use of systemic antibiotics holds promise for treatment of infected nonunions of long bones. It offers the advantage of sparing patients the complications and costs of prolonged intravenous antibiotics and is associated with satisfactory outcomes.
